# 6′-Sialylgalactose inhibits vascular endothelial growth factor receptor 2-mediated angiogenesis

**DOI:** 10.1038/s12276-019-0311-6

**Published:** 2019-10-11

**Authors:** Tae-Wook Chung, Eun-Yeong Kim, Hee-Jung Choi, Chang Woo Han, Se Bok Jang, Keuk-Jun Kim, Ling Jin, Young Jun Koh, Ki-Tae Ha

**Affiliations:** 1 Department of Korean Medical Science, School of Korean Medicine and Healthy Aging Korean Medical Research Center, Pusan National University, Yangsan, Gyeongnam 50612 Korea; 2 Department of Molecular Biology, College of Natural Sciences, Pusan National University, Geumjeong-gu, Busan 46241 Korea; 3 Department of Clinical Pathology, TaeKyeung University, Gyeongsan, Gyeongbuk 38547 Korea; 4 Department of Pathology, College of Korean Medicine, Dongguk University, Goyang, Gyeonggi-do 10326 Korea; 5 GI Innovation, Inc., A-1116, Tera Tower, Songpa-daero 167, Songpa-gu, Seoul 05855 Korea

**Keywords:** Drug development, Tumour angiogenesis

## Abstract

Angiogenesis should be precisely regulated because disordered neovascularization is involved in the aggravation of multiple diseases. The vascular endothelial growth factor (VEGF)-A/VEGF receptor 2 (VEGFR-2) axis is crucial for controlling angiogenic responses in vascular endothelial cells (ECs). Therefore, inactivating VEGFR-2 signaling may effectively suppress aberrant angiogenesis and alleviate related symptoms. In this study, we performed virtual screening, identified the synthetic disaccharide 6′-sialylgalactose (6SG) as a potent VEGFR-2-binding compound and verified its high binding affinity by Biacore assay. 6SG effectively suppressed VEGF-A-induced VEGFR-2 phosphorylation and subsequent in vitro angiogenesis in HUVECs without inducing cytotoxicity. 6SG also inhibited VEGF-A-induced extracellular-regulated kinase (ERK)/Akt activation and actin stress fiber formation in HUVECs. We demonstrated that 6SG inhibited retinal angiogenesis in a mouse model of retinopathy of prematurity and tumor angiogenesis in a xenograft mouse model. Our results suggest a potential therapeutic benefit of 6SG in inhibiting angiogenesis in proangiogenic diseases, such as retinopathy and cancer.

## Abnormal blood vessel formation: Synthetic disaccharide shows therapeutic promise

Therapy based on a synthetic molecule can block abnormal blood vessel formation, limiting the progression of diabetic eye conditions and tumor growth in mice. The growth of new blood vessels from existing vessels, called angiogenesis, is critical to wound healing and embryonic development. The main angiogenesis signalling pathway involves growth factors, including one called VEGFR-2. Disruption to this pathway plays a significant part in the development of multiple diseases. A South Korean team led by Ki-Tae Ha at Pusan National University, Yangsan, and Young Jun Koh at Dongguk University, Seoul, identified and trialed a synthetic disaccharide capable of binding to and limiting the activity of VEGFR-2 during faulty signaling. Trials on mice with the diabetic eye condition retinopathy, and mice with implanted tumors, showed that the compound inhibited excessive angiogenesis and limited disease progression.

## Introduction

Angiogenesis, the growth of new blood vessels from mature preexisting vessels, is an important process in various physiological conditions, such as embryonic development, wound healing, and tissue regeneration^1^, as well as in several pathological conditions, such as ischemic heart disease, rheumatoid arthritis, diabetic retinopathy, and tumor formation^2,3^, upon induction by the aberrant modulation of angiogenic cues. Vascular endothelial growth factor A (VEGF-A) and vascular endothelial growth factor receptor 2 (VEGFR-2) form the most crucial angiogenic signaling pathway, which coordinates the physiological and pathological growth of blood vessels, such as in the retina and in tumors^4,5^. Several lines of evidence support the usefulness of VEGF-A/VEGFR-2 inhibitors for treating various diseases related to aberrant angiogenesis^6^. Specifically, recent clinical achievements with the humanized anti-VEGF antibody bevacizumab^7^ have led to the development of related VEGF/VEGFR-2 inhibitors, including monoclonal antibodies^8^, kinase inhibitors^9^, and soluble decoy receptors^6,10,11^.

Natural compounds are vital in the control of human diseases and represent an important source of drug development as they provide structural and chemical diversity^12^. With advances in drug screening targeting aberrant signaling pathways, virtual screening has become a useful analytic modality for assessing the binding affinities of various natural compounds to defined target proteins and for predicting the biological efficacy of such candidates against their targets^13,14^. Many natural compounds selected through virtual screening that target VEGFR-2 and other tyrosine kinases have been developed and modified for better efficacy in terms of inhibiting abnormal angiogenesis^15,16^. Oleanolic acid was identified as a VEGFR-2-binding compound through virtual screening of a database of natural compounds and was shown to have inhibitory effects on aberrant angiogenic processes activated by the VEGF-A/VEGFR-2 axis^15^.

Human milk oligosaccharides (HMOs), encompassing over 130 different types, are the third-most abundant functional components in human milk^17^. HMOs have various biological functions, acting as prebiotics, antiadhesives, antimicrobials, and modulators of intestinal epithelial cells and immune cell functions^18,19^. Currently, sialylated HMOs have been shown to effectively inhibit angiogenesis and thereby reduce tumor growth by suppressing the VEGF-A/VEGFR-2 signaling axis^16^. Sialylgalactose moieties are commonly found at the nonreducing end of glycoproteins and glycolipids; sialylgalactose itself is not found in natural products^20^. Although such moieties were reported to inhibit influenza virus^21^, there are no previous reports on angiogenesis.

In this study, we sought to explore the improved antiangiogenic effects of 6′-sialylgalactose (6SG), a chemically synthesized sialylated galactose^20^ with higher binding affinity than other HMOs, in various in vitro and in vivo abnormal proangiogenic models, including retinopathy of prematurity (ROP) and cancer.

## Materials and methods

### Materials (chemicals and antibodies)

3′ sialylgalactose (3SG) and 6SG were purchased from Carbosynth Ltd. (Berkshire, UK). Recombinant human VEGF-A and VEGF-C were purchased from R&D Systems, Inc. (Minneapolis, MN). Antibodies against the phosphorylated or total forms of VEGFR-2, ERK, and Akt were purchased from Cell Signaling Technology (Danvers, MA). FITC-conjugated isolectin B4 (IB4) was purchased from Millipore-Sigma (St. Louis, MO). An antibody against PECAM1 was purchased from Agilent Dako (Santa Clara, CA). Antibodies against glyceraldehyde 3-phosphate dehydrogenase (GAPDH) and horseradish peroxide-conjugated secondary antibodies were purchased from Santa Cruz Biotechnology (Santa Cruz, CA).

### Simulation of protein-carbohydrate binding

A protein-small molecule docking method was used to predict whether 6SG, 6SL, and sialic acid interact with the extracellular domain of VEGFR-2 (PDB ID: 3S35). The three-dimensional structure of the VEGFR-2 IG3 domain was identified from the RCSB Protein Data Bank, and SwissDock was used to predict protein-ligand docking with 6SG, 6SL, and sialic acid and to obtain structure information.

### Expression and purification of VEGFR-2 IG3

A vector expressing the second and third IgG-like domains of VEGFR-2 (VEGFR-2 IG3) was constructed as described previously^22^. The vector was transformed into overexpression competent *Escherichia coli* BL21(DE3) cells. Each colony was inoculated in 5 ml of Luria Bertani (LB) medium enriched with 10 μg/ml kanamycin at 37 °C overnight. The cells were then incubated in 2 L of LB containing 10 μg/ml antibiotics at 37 °C until the OD_600_ reached 0.5–0.6. Next, VEGFR-2 IG3 expression was induced with 0.5 mM isopropyl-thio-β-d-galactopyranoside at 20 °C overnight, and the bacterial cells were then harvested by centrifugation at 3660 *×* *g* for 25 min at 4 °C. The cell pellets were resuspended in lysis buffer containing a protease inhibitor cocktail (Roche, Mannheim, Germany) and then sonicated (Branson Sonifier 450 sonicator; Danbury, USA). The cell suspensions were centrifuged at 20,170 *×* *g* for 45 min to separate the supernatant and pellet. The lysis process was repeated four times, and the final supernatant was concentrated using Vivaspin 20 and centrifuged at 1320 *×* *g*. Finally, the concentrated fractions of VEGFR-2 IG3 were purified by gel filtration chromatography using an equilibrated Superdex 200 10/300 GL fast protein liquid chromatography column (GE Healthcare, Sweden). Concentrated VEGFR-2 IG3 was analyzed by 15% sodium dodecyl sulfate polyacrylamide gel electrophoresis (SDS-PAGE).

### Surface plasmon resonance (SPR) biosensor analysis

The apparent dissociation constant (*K*_D_) between VEGFR-2 IG3 and 6SG or 6SL were measured using a Biacore T100 biosensor (GE Healthcare). The purified VEGFR-2 IG3 domain was covalently bound to the Series S sensor chip CM5 using an amine-coupling method as suggested by the manufacturer. A total of 150 μl of VEGFR-2 (50 μg/ml) in 10 mM sodium acetate (pH 5.0) was coupled via injection for 15 min at 10 μl/min, followed by injection of 1 M ethanolamine to deactivate residual amines. To measure kinetics at 25 °C, chemical compounds at concentrations ranging from 120 to 7.5 μM were prepared by dilution in HBS-EP^+^ buffer (10 mM HEPES, 150 mM NaCl, 3 mM EDTA, and 0.005% v/v surfactant P20) at pH 7.4. The immobilized ligand was regenerated by injecting 10 μl of 50 mM NaOH at a rate of 10 μl/min during the cycles.

### Cell culture

HUVECs were purchased from Cambrex Inc. (Walkersville, MD) and cultured in endothelial cell growth medium-2 (EGM-2, Cambrex Inc.). HUVECs at passage five to eight were used for experiments. Lewis lung carcinoma (LLC) cells were purchased from American Type Culture Collection (Manassas, VA). Mouse colon carcinoma (CT26) cells were provided by the Korean Cell Line Bank (Seoul, Korea). The cells were cultured in Dulbecco’s Modified Eagle Medium (DMEM, Thermo Fisher Scientific, Waltham, MA) supplemented with 10% fetal bovine serum (Sigma-Aldrich, St. Louis, MO, USA) and antibiotics (Gibco, Grand Island, NY). All cell lines were cultured at 37 °C in a cell culture incubator with 5% CO_2_.

### Cell viability assay

The cytotoxic effects of 3SG and 6SG were examined using an MTT labeling kit (Sigma-Aldrich). Briefly, HUVECs were cultured for 72 h in 24-well plates (Corning Inc.) in EBM-2 (Cambrex Inc.) containing 1% FBS at the indicated doses of HMOs and/or VEGF-A (50 ng/ml; R&D Systems Inc.). After removal of the culture medium, the cells were incubated with 300 μl of MTT (0.5 mg/ml) for 4 h at 37 °C in a 5% CO_2_ incubator. Formazan crystals in viable cells were dissolved in 300 μl of ethanol:DMSO (v/v, 1:1), and the absorbance in each well was measured at 540 nm using a SpectraMax M2 reader (Molecular Devices, Sunnyvale, CA).

### Western blot analysis

Western blot analysis was performed as previously described^16^. Briefly, HUVECs were treated with the indicated doses of 3SG and 6SG in the presence or absence of VEGF-A (50 ng/ml) for 30 min. Cells were then lysed using lysis buffer containing a protease inhibitor cocktail tablet (Roche) and processed for SDS-PAGE. The blots were incubated with the indicated primary antibodies. Pierce ECL plus reagent (Thermo Fischer Scientific) and ImageQuant LAS4000 (GE Healthcare) were used to detect protein bands.

### Tube-forming assay

To evaluate capillary-like tube formation by HUVECs, Matrigel-coated 24-well culture plates were used as previously described^23^. Matrigel (BD Bioscience, San Jose, CA) was added to each well of the 24-well culture plates, which were incubated at 37 °C for 1 h for polymerization. The cells were suspended in EBM-2 containing 1% FBS (1 × 10^4^ cells/well) and then added to the Matrigel-coated wells with or without 6SG at the indicated doses in the presence or absence of VEGF-A (50 ng/ml). The cells were further incubated for 15 h at 37 °C in 5% CO_2_. Each well of the culture plates was photographed with a Nikon Eclipse TS100 microscope (Nikon, Tokyo, Japan), and tube area was quantified using ImageJ software (NIH, Bethesda, MD).

### Migration assay

A HUVEC migration assay was performed using 24-well chambers containing polycarbonate membrane inserts (Corning Inc.) as previously described^23^. The cells were plated in the upper chamber with or without the indicated dose of 6SG, and EBM-2 containing 1% FBS with or without VEGF-A was added to the lower chamber. Cells were then allowed to migrate through the pores for an additional 24 h. Cells on the upper side of the membrane were removed using cotton swabs and stained with a Diff-Quik solution (Sysmex, Kobe, Japan). The membranes were photographed under a microscope (Nikon Eclipse TS100; Nikon). For quantitative analysis, the cells that migrated to the lower side of the membrane were counted. The results were calculated as the average number of migrated cells from three different membranes.

### Immunofluorescence

Immunofluorescence staining was performed as previously described^23^. Briefly, HUVECs were plated on 12 mm coverslips in 24-well tissue culture plates and treated at 37 °C for 24 h with or without 6SG at the indicated doses in the presence or absence of VEGF-A. The cells were immunostained with Texas Red®-X phalloidin (Invitrogen) for 30 min at room temperature. After several washes with PBS, the cells were analyzed under a fluorescence microscope (Zeiss AX10 Imager. M1; Carl Zeiss Microimaging, Oberkochen, Germany).

### Animals (mice and treatment)

Seven-week-old male C57BL/6 and BALB/c mice, inbred in a specific pathogen-free facility, were purchased from Orient Bio, Co. (Seongnam, Korea) and adapted for 1 week prior to the experiments. All animals were bred in a specific pathogen-free animal facility and had ad libitum access to normal chow (LabDiet, St. Louis, MO) and water. Animal care and experimental protocols were approved by the Animal Research Ethics Committee of Pusan University (approval No. PNU-2017-1603) and Dongguk University (approval No. IACUC-2016-050).

### Retinal angiogenesis assay

C57BL/6 mouse pups were injected intraperitoneally with the indicated doses of 6SG dissolved in saline at P4 and P5, and the retinas were harvested at P6.

### Oxygen-induced retinopathy

An Oxygen-induced retinopathy (OIR) mouse model was developed as reported previously^24,25^. Briefly, C57BL/6 mouse pups were exposed to 85% oxygen from P8 to P11 in a hypoxic/hyperoxic chamber with an O_2_ controller (Coy Laboratory Products, Grass Lake, MI), after which they were returned to room air for 5 days. 6SG dissolved in saline was administered intraperitoneally at P11 and P15 at the indicated doses, and the retinas were harvested at P16.

### Matrigel plug assay

Eight-week-old male C57BL/6 mice were injected subcutaneously with 500 μl of a BD Matrigel Matrix and heparin (50 unit/ml; BD Bioscience) mixture with or without the indicated doses of 6SG in the presence or absence of VEGF-A (100 ng/ml). Seven days after implantation, the Matrigel plugs were removed from the euthanized mice and fixed with formalin. The plugs were photographed with a digital camera (Nikon) and embedded in paraffin. Dissected tissues were stained with hematoxylin and eosin for microscopic observation. For quantification of the Matrigel plug assay results, the plugs were homogenized in 0.5 ml of distilled water and dissolved at 4 °C. Then, the homogenates were centrifuged, and the supernatant was incubated with 0.5 ml of Drabkin’s solution (Sigma-Aldrich) for 15 min at room temperature. Absorbance values were measured at 540 nm using a SpectraMax M2 reader (Molecular Devices).

### Tumor allograft

LLC and CT26 cells were suspended in PBS (1 × 10^6^ cells/100 μl) and subcutaneously inoculated into the right flanks of 8-week-old C57BL/6 (for LLC) and BALB/c mice (for CT26), which were randomly divided into three groups. The day after the injection of tumor cells, 6SG (0, 0.5 or 1.0 mg/kg) was administered intraperitoneally to mice once a day for 13 days. Tumor volume in LLC and CT26 tumor-bearing mice exposed to three doses of 6SG (0, 0.5 or 1.0 mg/kg) was measured on the indicated days (0, 8, 11, and 13) during the experiment. Tumor volume was measured with calipers and calculated according to the formula [(length × width)^2^/2]. All mice were euthanized 14 days after the inoculation of tumor cells, and the tumors were excised and weighed.

### Immunohistochemistry and morphometric analysis

Retinas were incubated with FITC-conjugated IB4 (Millipore-Sigma) and flat-mounted. All images were captured using a Nikon Eclipse Ts2 inverted fluorescence microscope equipped with a high-definition color camera (DS-Qi2, Nikon) and analyzed using NIS Elements Imaging Software (Nikon). To calculate the number of sprouts, IB4 staining was assessed in four 0.09 mm^2^ areas of the vascular front in each retina, and the values were averaged. Retina vascular density was determined by analyzing the density of IB4-positive areas based on the pixels in five randomly selected regions of interest located between an artery and a vein in each retina, and the values were averaged. Veins were identified based on blood vessel morphology, according to a previous paper^26^. In particular, veins surrounded by dense capillaries have a larger diameter than arteries during development. Retina vascular branch points and densities were determined by analyzing the density of IB4-positive areas based on the pixels in five randomly selected regions of interest located in areas of a vein in each retina, and the values were averaged. To determine the number of neovascular tufts (NVTs) in the OIR model, vascular tufts with diameters exceeding 50 μm were counted according to a previous study^27^. Tumors were immediately removed from euthanized tumor-bearing mice, fixed with formalin, and embedded in paraffin for immunohistochemical analysis. The paraffin sections were immunostained with an anti-PECAM1 antibody and visualized using a Dako EnVision kit (Dako). The tumor vascular density was determined by analyzing the density of PECAM1-positive areas based on the pixels in five randomly selected regions of interest.

### Statistical analysis

Differences between the mean values obtained for each experimental group were analyzed by one-way analysis of variance with Tukey’s post hoc test using GraphPad Prism (GraphPad Software, San Diego, CA). The minimum significance level was set at a *p* value of 0.05.

## Results

### Superior binding affinity of 6SG to VEGFR-2 and subsequent inhibition of VEGFR-2 phosphorylation in HUVECs

**Fig. 1:**
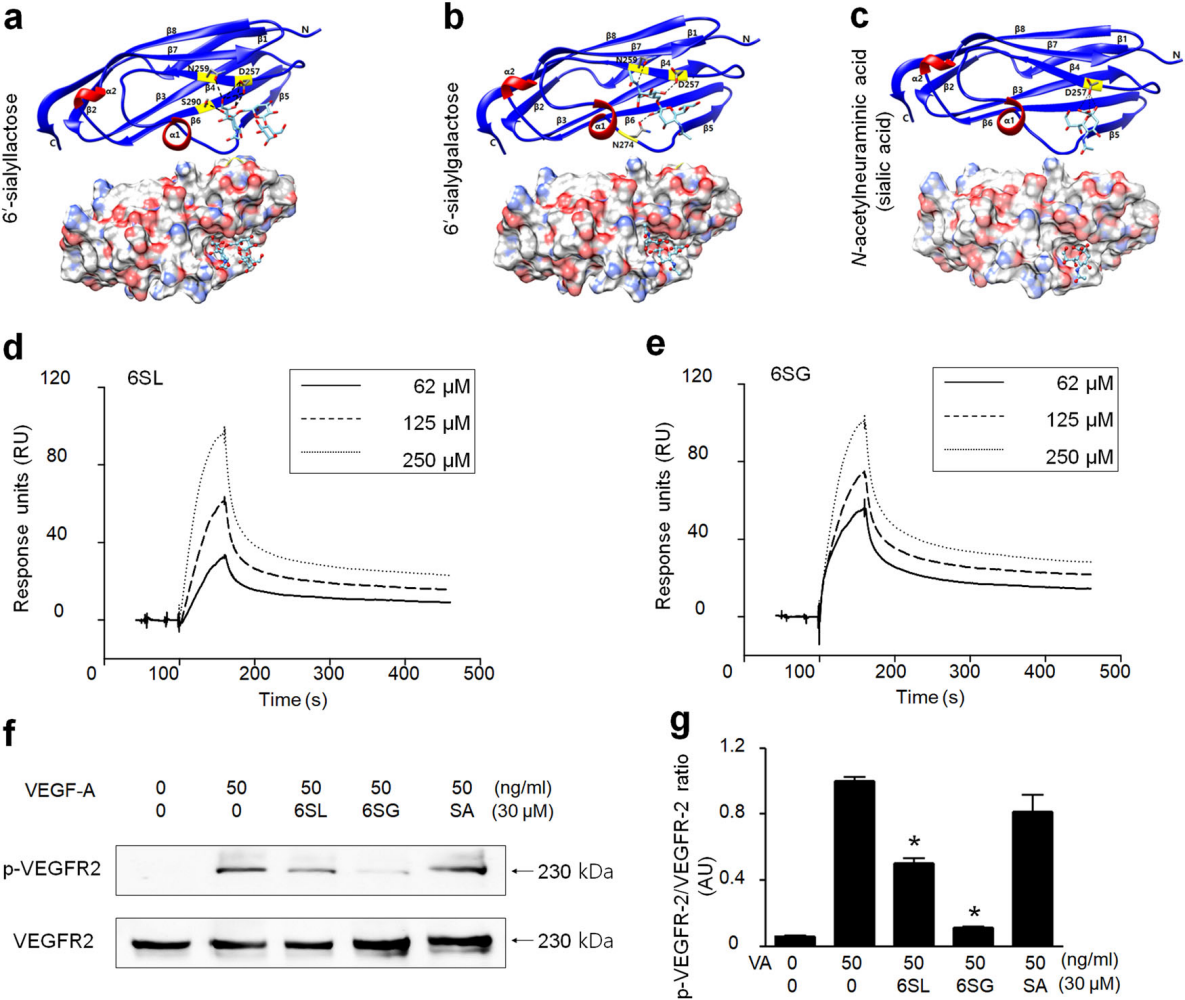
Screening milk sialic oligosaccharides for their ability to inhibit VEGF-induced VEGFR-2 phosphorylation. **a**–**c** Ribbon images of the VEGFR-2 structure bound to 6SG, 6SL, and *N*-acetylneuraminic acid (sialic acid) (upper row). Surface images of VEGFR-2 with HMOs in the pocket (stick model and space-filling model) showing carbon atoms (gray), oxygen atoms (red), nitrogen atoms (blue), and sulfur atoms (gold) (lower row). **d**, **e** Interactions of 6SG or 6SL with the second and third Ig-like domains of VEGFR-2 were measured using the Biacore assay. **f** HUVECs were treated with VEGF-A (50 ng/ml) and 6SL, 6SG, or SA (30 μM). VEGFR-2 phosphorylation (pVEGFR-2) was examined by western blot analysis. Total VEGFR-2 was used as a control. **g** Quantitative densitometric analysis of western blots **f**. The results represent the fold increase versus the positive control (second lane). The graph shows the mean ± standard deviation (SD; *n* = 3). ^*^P < 0.001 compared with the positive control

Using a protein-small molecule docking method, we identified 6SG, which interacted directly with the extracellular domain of VEGFR-2; the docking sites of 6SG were similar to those of 6′-sialyllactose (6SL) and sialic acid. 6SL bound to D257, N259, and S290 of the extracellular domain of VEGFR-2 IG3 (224–326) on one side of the binding pocket (Fig. 1a). Conversely, 6SG strongly interacted with three amino acids (D257, N259, and N274) in a triangle inside the binding pocket (Fig. 1b). 6SL was located in the exterior of the binding pocket more frequently than 6SG, and some parts of the ligand extended outside the pocket (Fig. 1a, b). In addition, sialic acid weakly bound to D257 only (Fig. 1c).

To validate the binding affinity between chemical ligands and VEGFR-2, we performed a Biacore assay. Compared with the reference HMO, 6SL (*K*_D_ = 3.05 nM), 6SG had a slightly higher binding affinity with the purified second and third IgG-like domains of VEGFR-2 (*K*_D_ = 2.35 nM; Fig. 1d, e).

We next examined whether 6SG has stronger inhibitory effects on VEGFR-2 activity than other HMOs. 6SG had the most potent inhibitory effect on VEGF-A-induced phosphorylation of VEGFR-2 in HUVECs following treatment with VEGF (50 ng/ml) for 30 min with or without pretreatment with 30 μM HMOs (Fig. 1f, g). 6SG inhibited VEGFR-2 phosphorylation by approximately 85%, whereas 6SL and SA inhibited VEGFR-2 phosphorylation by approximately 50 and 15%, respectively (Fig. 1g). These results indicate that 6SG inhibited VEGF-A-induced VEGFR-2 activation in HUVECs more effectively than other HMOs.

Taken together, these results indicate that 6SG functions as a strong inhibitor of VEGFR-2 by stably binding to the negatively charged D257 residue and the polar N259 and N274 residues.

### 6SG suppresses VEGFR-2 phosphorylation in HUVECs more effectively than 3SG

**Fig. 2:**
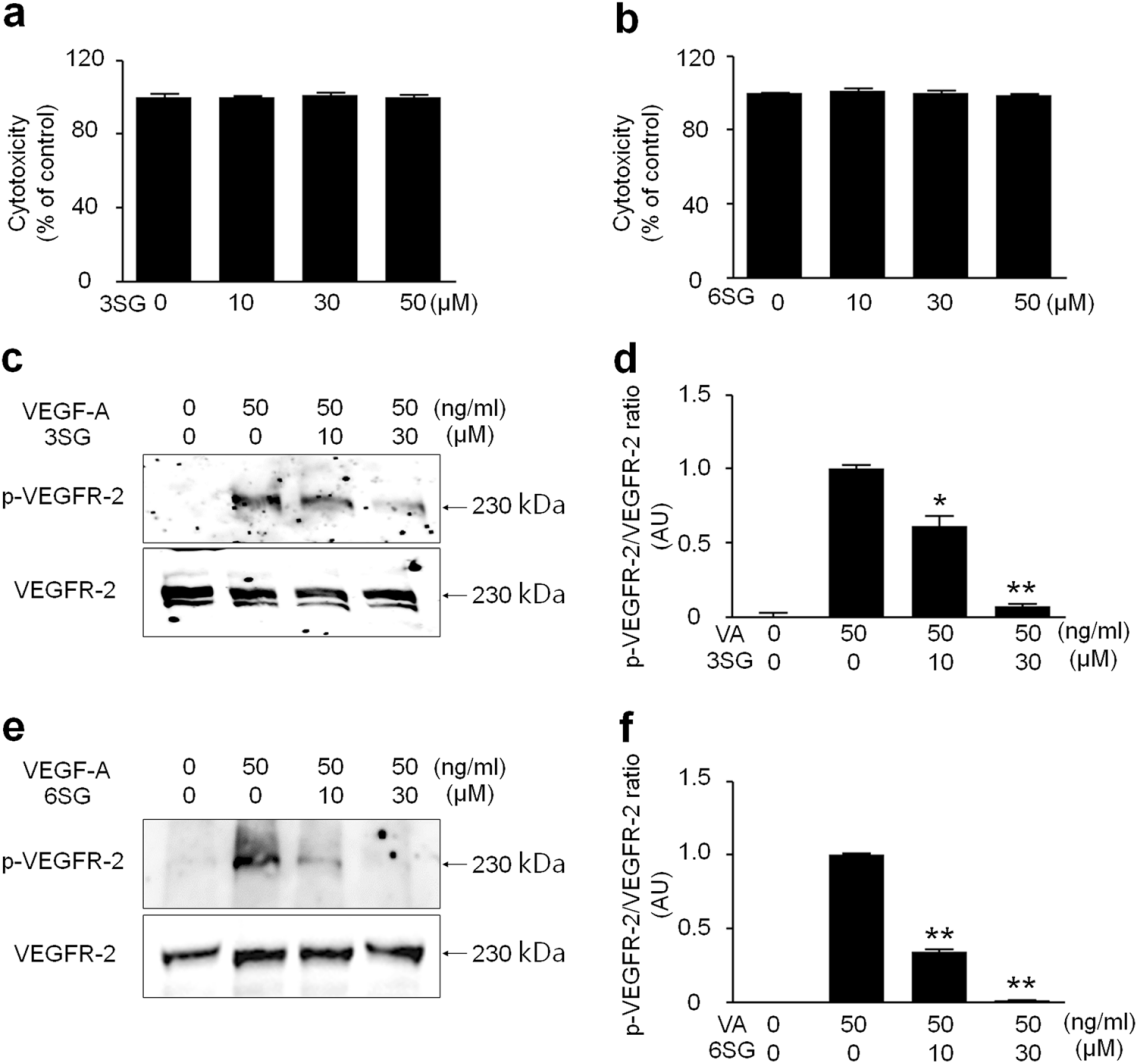
Effects of 3SG and 6SG on VEGF-induced VEGFR-2 phosphorylation. **a**, **b** HUVECs were treated with the indicated doses of 3SG or 6SG for 72 h. The cytotoxicity of 3SG and 6SG in HUVECs was measured by MTT assays, and the results are presented as the mean ± SD. **c**, **e** HUVECs were treated with VEGF-A and/or 3SG and 6SG at the indicated doses. pVEGFR-2 was examined by western blot analysis. Total VEGFR-2 was used as a control. **d**, **f** Quantitative densitometric analysis of western blots in **c** and **e**. The results represent the fold increase versus the positive control (second lane). The graph shows the mean ± SD (*n* = 3). ^*^P < 0.01 and ^**^P < 0.001 compared with the positive control

To examine the cytotoxicity of 3SG and 6SG, HUVECs were treated with varying concentrations (up to 50 μM) of both HMOs for 48 h, and cell viability was subsequently evaluated by MTT assays. Neither 3SG nor 6SG caused significant cytotoxicity in HUVECs at any tested dose (Fig. 2a, b). We next determined whether 3SG and 6SG inhibit VEGF-A-induced phosphorylation of VEGFR-2 in HUVECs. Pretreatment of HUVECs with different doses of 3SG or 6SG (10 and 30 μM) prior to VEGF-A treatment (50 ng/ml) for 30 min revealed that both HMOs inhibited the phosphorylation of VEGFR-2 in a dose-dependent manner (Fig. 2c–f). At 10 μM, 3SG and 6SG inhibited VEGFR-2 phosphorylation by ~40% and 60%, respectively, showing that 6SG inhibited VEGF-A-induced phosphorylation of VEGFR-2 more effectively than 6SG (Fig. 2e, f).

### 6SG inhibits VEGF-induced growth, tube formation, migration, and actin stress fiber formation in HUVECs

**Fig. 3:**
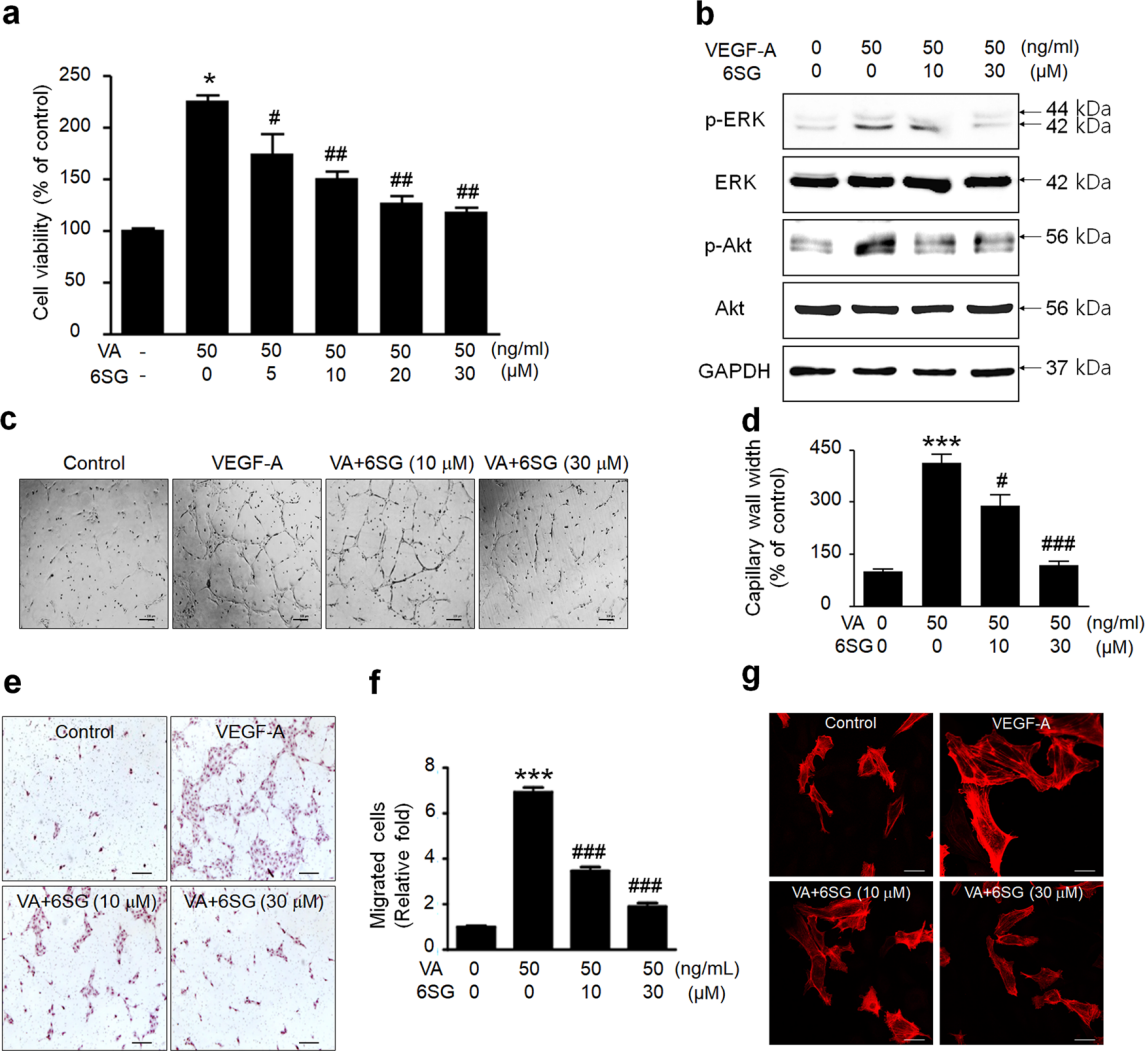
Effects of 6SG on VEGF-induced signaling and angiogenic features in HUVECs. **a** HUVECs were treated with VEGF-A and/or 6SG at the indicated doses. The effect of 6SG on the viability of VEGF-A-treated HUVECs was measured by MTT assays. The graph shows the mean ± SD. ^*^P < 0.001 compared with the negative control (first lane), ^#^*P* < 0.05 and ^##^*P* < 0.001 compared with the positive control (second lane). **b** HUVECs were treated with VEGF-A and/or 6SG at the indicated doses. The levels of ERK (p-ERK) and Akt (p-Akt) phosphorylation were examined by western blot analysis. Total ERK, total Akt, and GAPDH were used as controls. **c** Representative images of tube formation by HUVECs treated with VEGF-A (50 ng/ml) and/or 6SG at the indicated doses. **d** Quantitative analysis of tube length (mm) **c**. The graph shows the mean ± SD. ^*^P < 0.01 compared with the control (first lane); ^#^*P* < 0.01 compared with the positive control (second lane). **e** Representative images of HUVEC migration following tr**e**atment with VEGF-A (50 ng/ml) and/or 6SG at the indicated doses. The migrated cells were stained with hematoxylin and eosin. **f** Quantitative analysis of migrated HUVECs **e**. The graph shows the mean ± SD. ^*^P < 0.01 compared with the control (first lane); ^#^*P* < 0.01 compared with the positive control (second lane). **g** Representative images of actin stress fibers in HUVECs treated with VEGF-A (50 ng/ml) and/or 6SG at the indicated doses. Actin stress fibers were stained with phalloidin (red)

To determine the inhibitory effect of 6SG on VEGF-A-induced HUVEC viability, these cells were treated with VEGF-A (50 ng/ml) for 30 min with or without 6SG pretreatment (10 or 30 μM) for 48 h, and cell viability was evaluated by MTT assays (Fig. 3a). 6SG significantly and dose-dependently inhibited VEGF-induced HUVEC growth (Fig. 3a). In addition to the inhibitory effect of 6SG on VEGFR-2 phosphorylation, 6SG dose-dependently inhibited the activation of downstream VEGFR-2 signaling, including the phosphorylation of ERK and Akt (Fig. 3b), which are related to VEGF-A-induced proliferation, migration, tube formation, and actin filament formation in HUVECs. VEGF-A-induced tube formation by HUVECs was consistently and dose-dependently inhibited by 6SG treatment (Fig. 3c, d). Furthermore, VEGF-A-induced HUVEC migration was markedly decreased upon 6SG treatment (Fig. 3e, f). We also observed that 6SG effectively inhibited the formation of VEGF-A-induced actin filaments in HUVECs, as shown by filamentous arrays stained with phalloidin, which contribute to cell migration (Fig. 3g).

### 6SG inhibits developmental and pathologic angiogenesis in the mouse retina

**Fig. 4:**
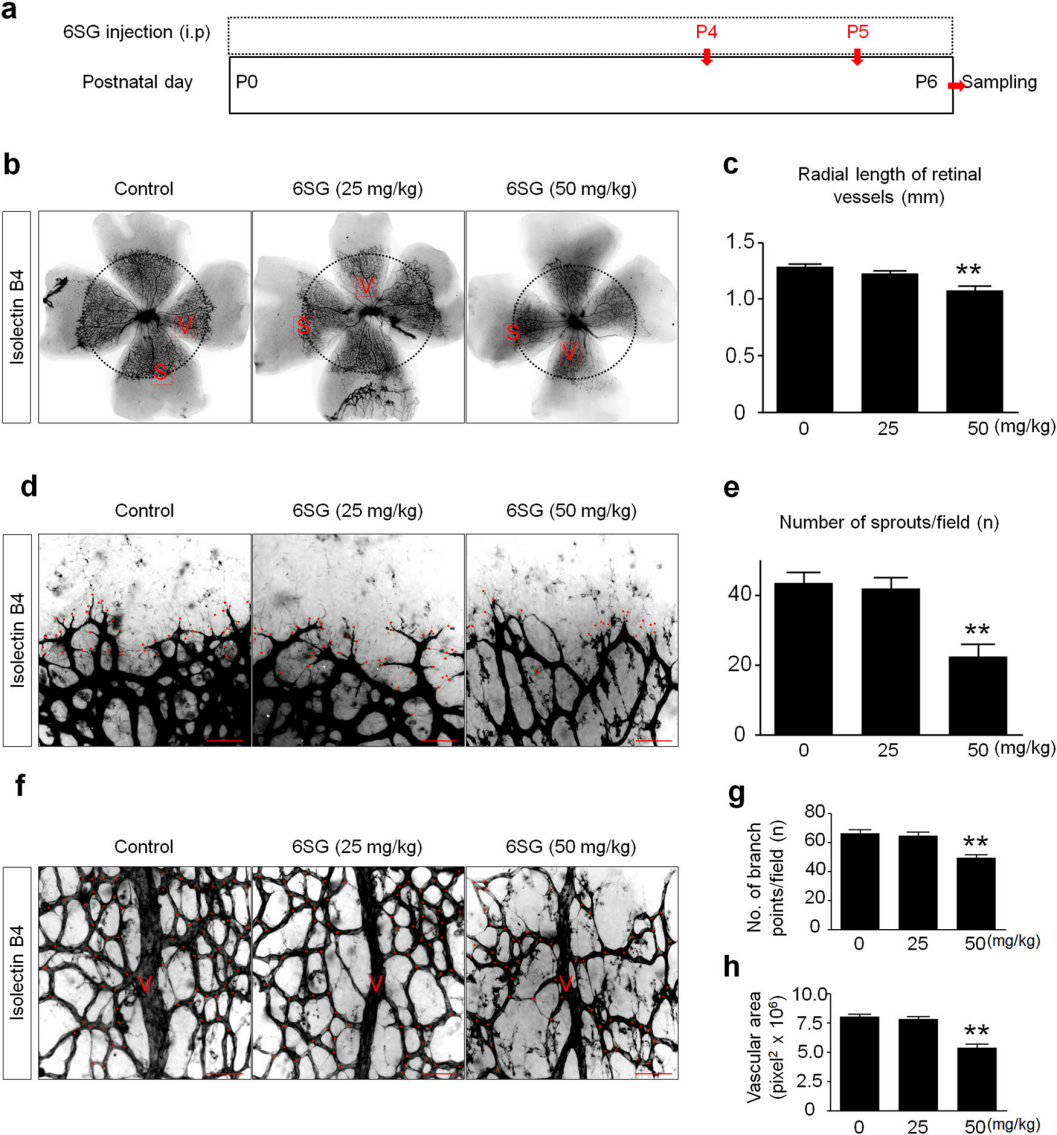
Effect of 6SG on in vivo models of developmental angiogenesis in the mouse retina. **a** Diagram depicting the experimental schedule for the generation of in vivo models of developmental angiogenesis in the mouse retina and 6SG treatments. **b** Representative tiled images of retinal vasculature at two doses of 6SG (25 or 50 mg/kg). The radial lengths are drawn with dotted black lines. “**S** (sprout)” in red squares is magnified in **d**. “**V** (vein)” in red squares is magnified in **f**. Scale bar, 500 μm. **c** Quantitative analysis of radial length **b**. The graph shows the means ± SD (*n* = 5). ^**^P < 0.01 compared with the control. **d** Representative magnified images of “S” in **b** (sprout formation) following treatment with 6SG (25 or 50 mg/kg). The sprouts are marked with red dots. Scale bar, 50 μm. **e** Quantitative analysis of sprout number **d**. The graph shows the mean ± SD (*n* = 5). ^**^P < 0.01 compared with the control. **f** Representative magnified images of “V” in **b** (retinal vasculature) following treatment with 6SG (25 or 50 mg/kg). Branch points are marked with red dots. Scale bar, 50 μm. **g**, **h** Quantitative analysis of the number of branch points and the vascular area **f**. The graph shows the mean ± SD (*n* = 5). ^**^P < 0.01 compared with the control

**Fig. 5:**
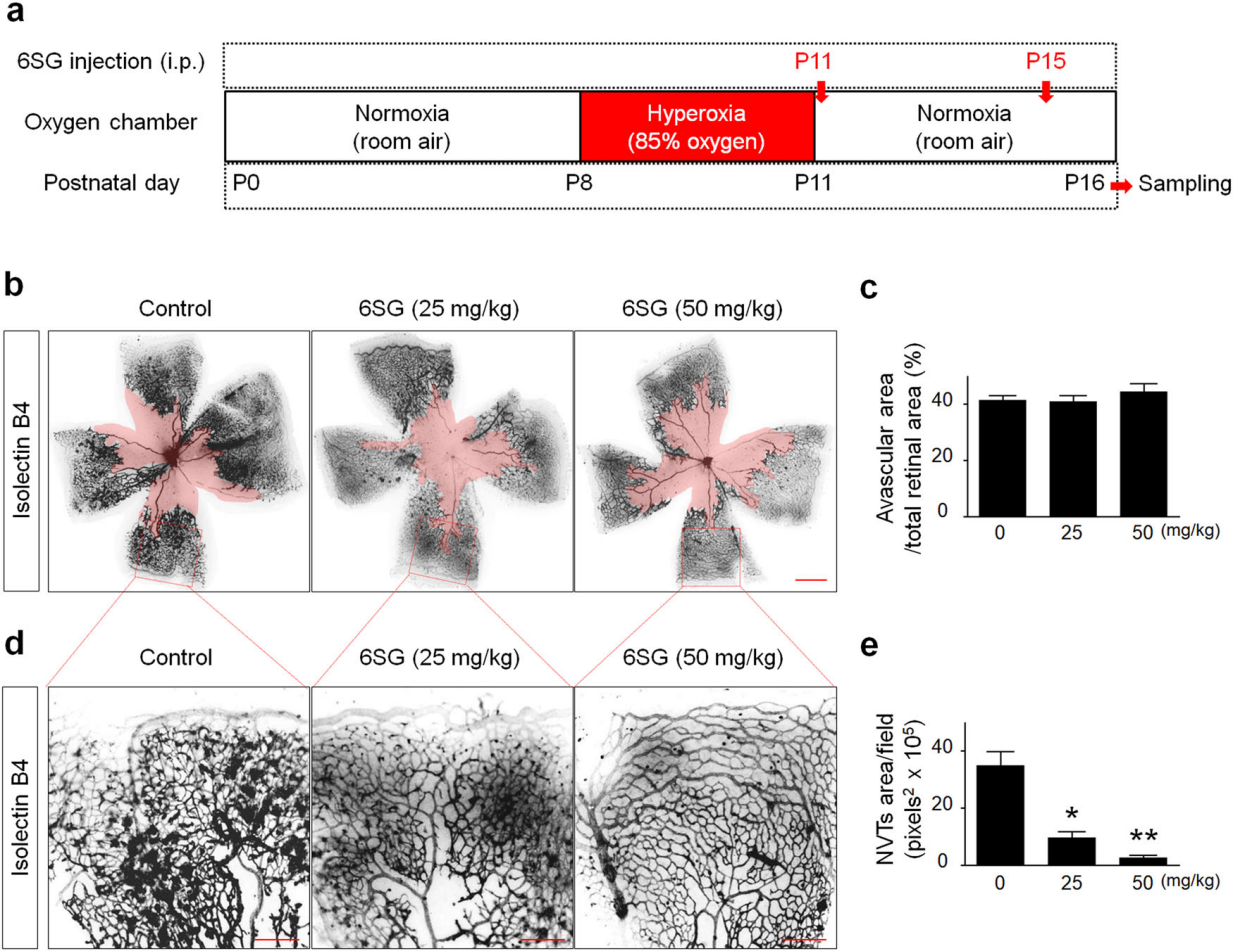
Effect of 6SG on in vivo models of pathologic angiogenesis in the mouse retina. **a** Diagram depicting the experimental schedule for the generation of the OIR model and 6SG treatments. **b** Representative tiled images of retinal vasculature after two doses of 6SG (25 or 50 mg/kg). The avascular areas are drawn with light red. Scale bar, 500 μm. **c** Quantitative analysis of the avascular area **b**. The graph shows the mean ± SD (*n* = 5). **d** Representative images of vasculature in OIR mouse retinas following treatment with 6SG (25 or 50 mg/kg). Scale bar, 200 μm. **e** Quantitative analysis of NVT area **d**. The graph shows the mean ± SD (*n* = 5). ^*^P < 0.05 and ^**^P < 0.01 compared with the control

To investigate whether 6SG inhibits retinal angiogenesis in mice, we administered 6SG to postnatal C57BL/6 mice at two doses (25 and 50 mg/kg) (Fig. 4a). The high-dose 6SG treatment group showed a significantly shorter radial length of retinal vessels than the control group (Fig. 5b, c). There were significantly fewer sprouts at the front of retinal blood vessels following high-dose 6SG treatment compared with the control (Fig. 4d, e). Consistently, the high-dose 6SG treatment group presented significantly fewer branch points and lower vascular density in the retina than the control group (Fig. 4f–h). These results indicate that 6SG effectively inhibits angiogenesis in the retina during development.

To determine whether 6SG inhibits pathologic retinal angiogenesis in mice, we generated an oxygen-induced retinopathy (OIR) mouse model and injected 6SG at two doses (25 and 50 mg/kg) (Fig. 5a). We then measured the avascular area and number of NVTs (Fig. 5b–e). There were no significant differences in the avascular regions of the retina between the control and 6SG treatment groups, regardless of dose (Fig. 5b, c). These results indicate that 6SG inhibits pathologic angiogenesis in the retinas of OIR mice.

### 6SG inhibits in vivo angiogenesis in Matrigel plugs and implanted tumors

**Fig. 6:**
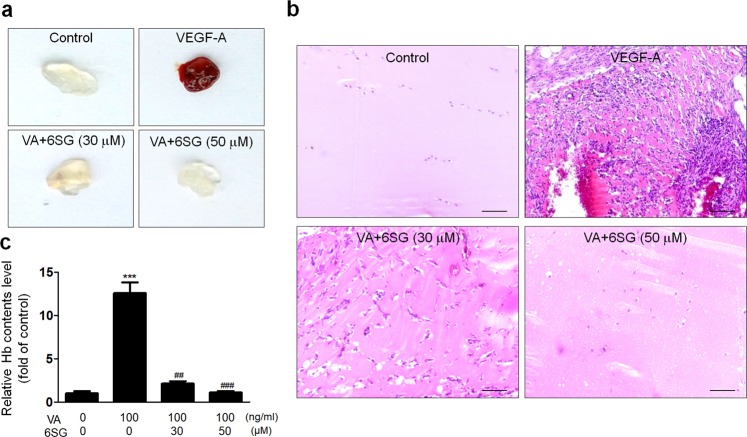
Inhibitory effect of 6SG in VEGF-induced in vivo neovascularization. **a** Representative photographs and **b** histological images of Matrigel plugs in mice treated with VEGF-A (100 ng/ml) and/or 6SG at the indicated doses. **c** Quantification of functional vasculature inside the Matrigel plugs by measuring Hb content using Drabkin’s reagent. The graph shows the mean ± SD. ^***^P < 0.001 compared with the control (first lane); ^##^*P* < 0.01 and ^###^*P* < 0.001 compared with the positive control (second lane)

**Fig. 7:**
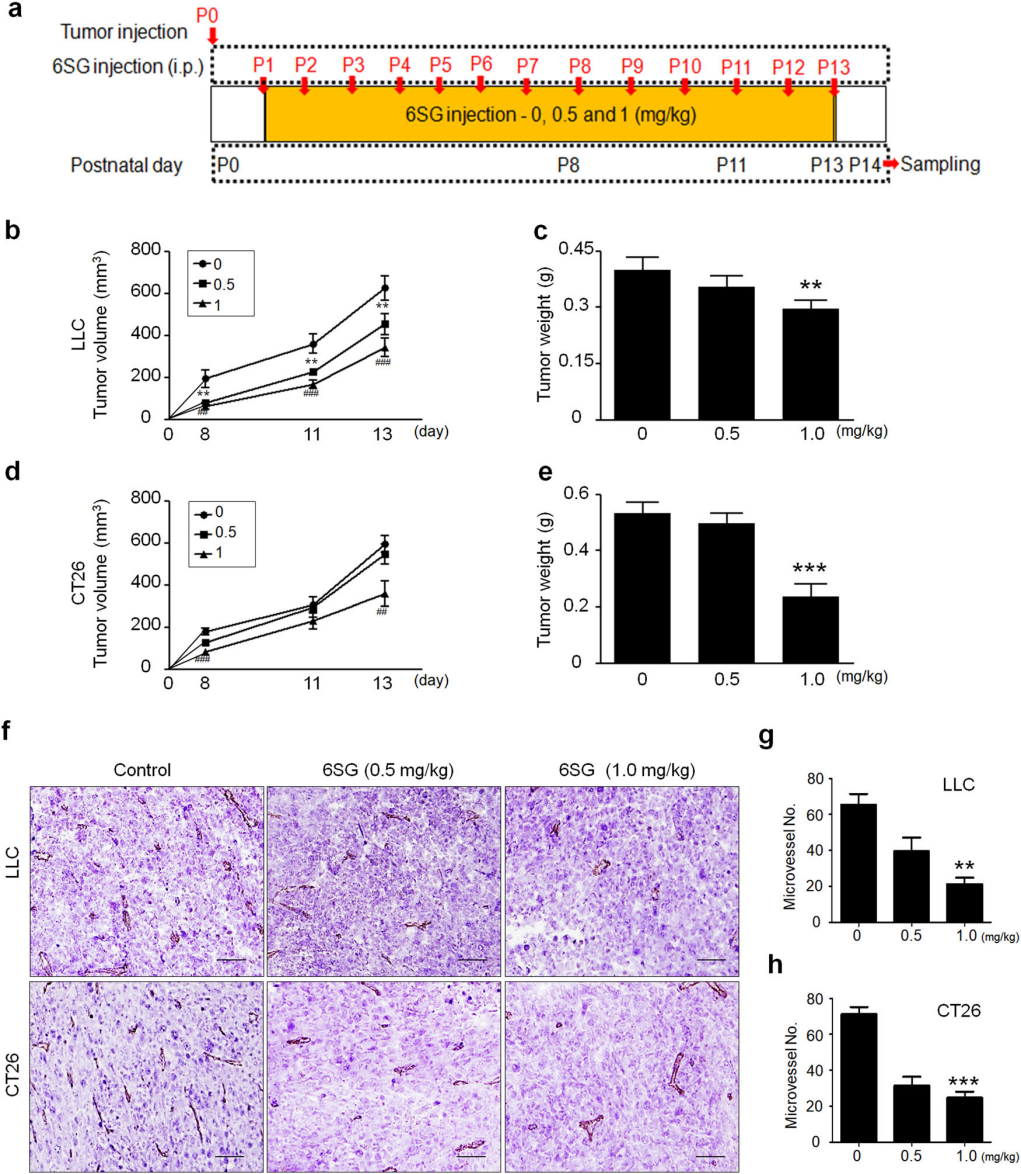
Effect of 6SG on the suppression of tumor growth and angiogenesis in tumor-bearing mice. **a** Diagram depicting the experimental schedule for growth inhibition of allograft tumors by 6SG. **b**, **d** Tumor volume in LLC and CT26 tumor-bearing mice after treatment with 6SG (0, 0.5 or 1.0 mg/kg) on the indicated days (day 0, 8, 11, and 13). **c**, **e** The weights of tumors resected from LLC and CT26 tumor-bearing mice on day 14 after treatment with 6SG (0.5 or 1.0 mg/kg). The graph shows the mean ± SD. ^**^P < 0.01 and ^***^P < 0.001 compared with the control. **f** Representative images of PECAM1^+^ vasculature in LLC and CT26 tumors in mice treated with three different doses of 6SG (0, 0.5 or 1.0 mg/kg). Scale bar, 100 μm. **g**, **h** Quantitative analysis of the vasculature. The graph shows the mean ± SD. ^**^P < 0.01 and ^***^P < 0.001 compared with the control

To elucidate the inhibitory effects of 6SG on tumor angiogenesis in vivo, a Matrigel plug assay was performed. VEGF-A-induced vascularization in Matrigel was effectively hindered by 6SG treatment (Fig. 6a–c). In addition, to investigate whether 6SG inhibits tumor growth and angiogenesis, we administered three different doses (0, 0.5 or 1.0 mg/kg) of 6SG to LLC and CT26 tumor-bearing mice. Then, we measured tumor volume and weight and assessed PECAM1^+^ vasculature (Fig. 7). Tumor volume and weight were significantly lower in the high-dose 6SG treatment group than in the control group (Fig. 7b–e). Consistently, in both the LLC and CT26 groups, 6SG treatment significantly reduced the PECAM1^+^ vascular density in tumors compared with the control (Fig. 7f–h).

## Discussion

Disordered angiogenesis aggravates relevant diseases; therefore, angiogenesis should be carefully regulated^2,3^. The VEGF/VEGFR-2 axis is crucial for controlling angiogenic responses in ECs^4,5^. Modulating the VEGF/VEGFR-2 axis is therefore effective for coordinating angiogenesis and vascular functions to alleviate various disease symptoms^6^. Recent studies have shown that anti-VEGF agents, such as monoclonal antibodies^7,8^, kinase inhibitors^9^, and soluble decoy receptors^10,11^, effectively inhibit angiogenesis and consequently suppress symptoms of diseases, such as ROP and cancer^2^. Although protein-based drugs are superior to small molecules such as oligosaccharides in terms of target specificity and safety, their clinical use is limited due to costly production, the risk of immunogenicity with long-term treatment, and limited access to targeted pathologic sites due to their large molecular size^28^. Natural oligosaccharides, such as HMOs, have a low molecular weight, low immunogenicity, and high accessibility to therapeutic targets, leading us to develop HMOs with inhibitory effects on the VEGF-A/VEGFR-2 axis. A sialylated HMO, 3SL, was recently reported to inhibit angiogenesis by suppressing VEGFR-2 signaling^16^. Therefore, we aimed to develop superior oligosaccharides with higher binding affinities, which may result in improved antiangiogenic effects.

Docking between natural compounds and their target proteins has indicated the potential for inhibiting signal transduction by the target protein^29,30^. Thus, evaluating the interactions between candidate molecules and their target proteins through virtual screening prior to in vitro or in vivo analysis can greatly facilitate drug discovery and biomedical research^15,16^. Oleanolic acid, a natural compound, was previously identified as a candidate VEGFR-2 inhibitor through virtual screening; several in vitro and in vivo assays verified that oleanolic acid is an effective inhibitor of VEGFR-2^15^. In a previous study, we performed virtual docking screening and identified a potent HMO, 3SL, that strongly interacts with VEGFR-2^16^. Here, we demonstrated that 6SG interacted with the extracellular domain of VEGFR-2 in the triangle inside the binding pocket; in contrast, 6SL interacted with more residues outside the binding pocket compared with 6SG, which led to a weaker binding affinity for VEGFR-2. Consequently, we speculated that a synthetic disaccharide, 6SG, may function as a potent inhibitor of VEGFR-2, with greater efficacy than other natural HMOs. Subsequent Biacore analysis showed that 6SG strongly interacted with the second and third IgG-like domains of VEGFR-2, which are the VEGF-binding sites necessary for its phosphorylation; accordingly, VEGF-induced growth of HUVECs and VEGFR-2 phosphorylation in HUVECs were suppressed more effectively by 6SG than by 3SG. Moreover, we observed that 6SG effectively inhibited the activation of VEGFR-2 and its downstream signaling pathways, including the phosphorylation of ERK and Akt (Fig. 3b). As such, 6SG effectively inhibited features of VEGF-induced angiogenesis, including tube formation and cell migration. As VEGF induces actin stress fiber formation in endothelial cells^31^, 6SG suppressed the VEGF-provoked stretch of actin filaments in HUVECs (Fig. 3c–g). These studies suggest that 6SG regulates angiogenesis in the early stage to induce endothelial cell permeability, proliferation and migration via destabilization of endothelial cell-cell junctions and endothelial cell-basement membrane interactions.

In addition, we found that 6SG did not suppress VEGF-C-induced VEGFR-3 phosphorylation in HUVECs at the concentrations used to inhibit VEGFR-2 (Supplementary Fig. S1). These results demonstrate that 6SG effectively and specifically binds to the receptor binding site of VEGFR-2 and therefore inhibits angiogenesis by suppressing VEGF signaling pathways in ECs.

ROP is an important ocular disease that frequently occurs in preterm infants. Because of the complicated pathology of ROP development, trials investigating the prevention and treatment of ROP have shown limited efficacy^32,33^. Anti-VEGF therapies have been used for the treatment of diseases involving aberrant angiogenesis. Patients with ophthalmologic diseases, including ROP, have been treated with anti-VEGF therapies, and positive outcomes have been reported^32–34^. Therefore, we examined whether 6SG affects retinal angiogenesis; we observed that 6SG inhibited retinal angiogenesis during mouse retinal development, as demonstrated by the decreased sprout count in the front of the tip cells and the decreased vascular density (Fig. 4a–e). In addition, 6SG inhibited aberrant retinal angiogenesis in the mouse OIR model, an animal model of ROP, as demonstrated by the suppressed formation of NVTs (Fig. 4f, g). These effects partially explain why ROP is less prevalent in breastfed infants than in formula-fed infants^35,36^.

Following Judah Folkman’s claims that tumors require blood vessels for survival and growth and that tumors could be starved by cutting off the vessels, antiangiogenic research has been actively conducted for the past 40 years^37^. Folkman’s hypothesis thus influenced the development of antiangiogenesis drugs, particularly anti-VEGF-A agents such as bevacizumab^7^. Bevacizumab is now approved as a first-line treatment for non-small cell lung cancer (NSCLC) and as a first- or second-line treatment in combination with 5-fluorouracil chemotherapy for metastatic colorectal cancer^7,12,37–39^. To date, most studies have focused on controlling the VEGF-A/VEGFR-2 axis to alleviate diseases such as retinopathy and cancer;^6^ therefore, we determined whether 6SG also affects tumor angiogenesis using mouse models of NSCLC and colorectal cancer. 6SG effectively inhibited tumor growth (Fig. 7a–d, Supplementary Fig. S2) and angiogenesis in LLC and CT26 tumor-bearing mouse models (Fig. 7e–g). Notably, due to the inhibitory effect of 6SG on VEGFR-2, we suggest that tumor growth was hindered by 6SG treatment. However, several recent studies have revealed that antiangiogenic therapy is not always beneficial for treating cancer because excessive blockade of VEGFR2 signaling activates the infiltration of immunosuppressive innate immune cells, such as Ly6C^low^ monocytes, which in turn promote tumor growth^40–42^. Therefore, the antitumor efficacy of 6SG in treating refractory tumors could potentially be maximized by combination with agents targeting tumor-infiltrating immunosuppressive monocytes.

In conclusion, we showed that 6SG, a potent VEGFR-2-binding HMO identified by virtual screening, binds to the extracellular domain of VEGFR-2 and effectively inhibits the activation of VEGFR-2 and its downstream signals in HUVECs. As a result, 6SG inhibits physiological and pathological features of angiogenesis, such as retinal and cancer angiogenesis, as well as tubule formation and migration by HUVECs, thereby reducing the characteristics of ROP and the growth of allograft tumors. Our results suggest that 6SG may be a useful novel antiangiogenic agent.

## Supplementary information

Supplemtary material
