# Supplementary material for: 6′-Sialylgalactose inhibits vascular endothelial growth factor receptor 2-mediated angiogenesis
Source: Exp Mol Med. 2019 Oct 11;51(10):120. doi: 10.1038/s12276-019-0311-6 (PMC6802645; doi:10.1038/s12276-019-0311-6)
Supplement: Supplementary file 1 — Supplemtary material [file 12276_2019_311_MOESM1_ESM.docx]

**Supplementary materials**


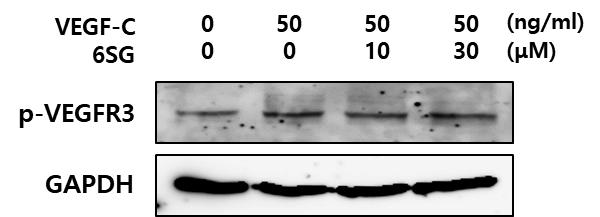


**Supplementary Fig. S1. Effects of 6SG on VEGF-C-induced VEGFR3 phosphorylation.** HUVECs were treated with VEGF-C and/or 6SG at the indicated doses. Levels of VEGFR-3 phosphorylation (pVEGFR-3) were examined by western blot analysis. GAPDH was used as a control.

**
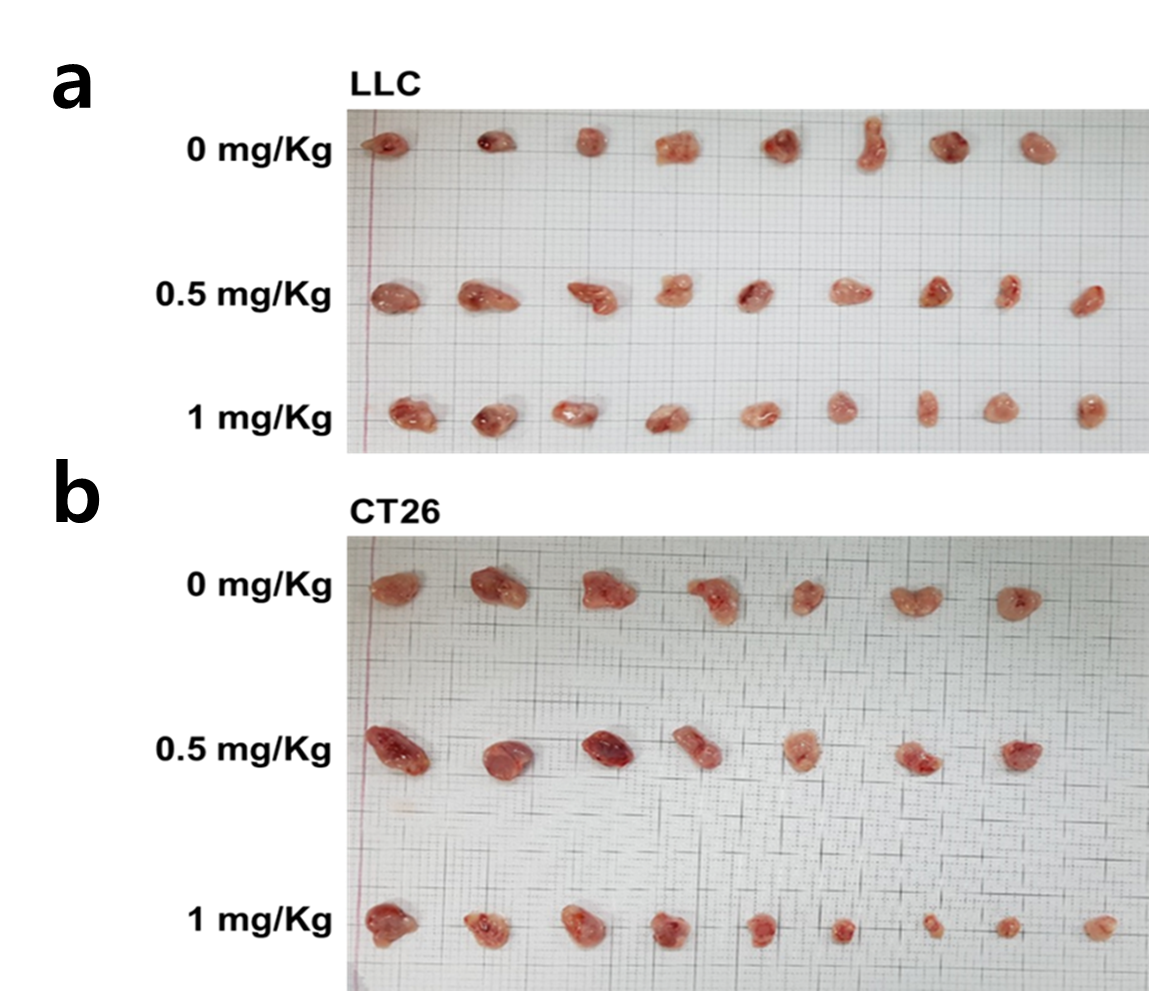
**

**Supplementary Fig. S2. Suppression of tumor growth by 6SG in tumor-bearing mice. a** LLC cells (1 × 10^6^/100 μl in PBS) and **b** CT26 colon carcinoma cells (1 × 10^6^/100 μl in PBS) were subcutaneously inoculated into back of mice. The next day after inoculation, indicated dosages of 6SG were injected to mice daily. At the end of experiment, the tumors were picked out from sacrificed mice, and pictures were taken.
